# Generalized anxiety disorder among mothers attending perinatal services during COVID-19 pandemic: using ordinal logistic regression model

**DOI:** 10.1016/j.heliyon.2022.e09778

**Published:** 2022-06-22

**Authors:** Mesfin Esayas Lelisho, Amanuel Mengistu Merera, Seid Ali Tareke, Sali Suleman Hassen, Sebwedin Surur Jemal, Admasu Markos kontuab, Meseret Mesfin Bambo

**Affiliations:** Department of Statistics, College of Natural and Computational Science, Mizan-Tepi University, P.O. Box 121, Tepi, Ethiopia

**Keywords:** Generalized anxiety disorder, Mental health, Prenatal period, COVID-19

## Abstract

**Background:**

Generalized anxiety disorder is characterized by excessive and uncontrollable worry about a variety of events. It is critical to ensure a pregnant mother's mental health in order to reduce pregnancy and birth-related problems. The major goal of current study was to identify the factors associated with generalized anxiety disorder among mothers attending perinatal services in the study area during COVID-19 using ordinal logistic regression.

**Methods:**

The institution-based cross-sectional study was conducted from July 10^th^, 2020 to August 10^th^, 2020 at Kembata Tembaro zone, Southern Ethiopia. The current study included 423 mothers. The GAD-7 scale was used to assess the anxiety level among mothers. An Ordered logit model was used to identify the determinants of GAD. Brant test of the parallel line was utilized to check proportionality assumption. The statistical significance was determined using an adjusted proportional odd ratio with a 95%CI, and a p-value <5%. STATA software version 14 was used to analyze statistical data.

**Results:**

Of all 423 mothers attending perinatal service during COVID-19; 134(31.7%), 171(40.4%), 85(20.1%), and 33 (7.8%) had non/minimal to severe generalized anxiety disorder respectively. The results of multivariable proportional odds model (POM) showed that the variables town residents [aPOR = 1.827; 95% CI:1.233–2.708], having alcohol habit [aPOR = 3.437, 95% CI = 1.397–8.454], having occupation [aPOR = 0.509, 95% CI: 0.303–0.857], being health care worker [aPOR = 0.117, 95% CI = 0.044–0.311], having chronic illness [aPOR = 7.685, 95% CI = 3.045–19.39], having family history of anxiety/mood disorder [aPOR = 7.839, 95% CI = 2.656–23.12], fear of contracting COVID-19 [aPOR = 1.704, 95% CI = 1.152–2.521], having moderate social support [aPOR = 0.648, 95% CI = 0.425–0.989], having strong social support [aPOR = 0.495, 95% CI = 0.272–0.901] were significantly associated with generalized anxiety disorder at 5% level of significance.

**Conclusion:**

Current findings concluded that the prevalence of GAD among mothers attending perinatal service during COVID-19 was high. The covariates like being town resident, lower-income status, occupation status, having a chronic illness, having a positive family history of anxiety or mood disorder, perceived social support, and fear of the COVID-19 were significantly associated with generalized anxiety disorder among mothers. Mothers who visit perinatal services should be given special consideration to improve health care services and ensure their mental health.

## 1 Introduction

Generalized anxiety disorder (GAD) is the common and long-lasting psychiatric condition which is marked by excessive worrying, anxiety, tension, and physical symptoms [1, 2]. It is also characterized by excessive anxiety and worries about everyday life events for no apparent reason [3]. GAD mostly affects women, especially those during childbearing age [4, 5]. During the pandemic period, studies on mothers attending perinatal service revealed that mental health problems were on the rise in this population [6, 7, 8]. Furthermore, a mental health of mother's during the perinatal period is critical for avoiding preterm birth and complications during delivery [9, 10].

The perinatal period is well recognized as a sensitive time in a woman's life, and anxiety symptoms are common during this time [11]. The perinatal period of a woman lasts from 22 weeks of pregnancy to one week after birth, and it is during this time that the majority of pregnancy and birth-related issues arise due to a variety of physiological and environmental factors [12]. Pregnant women are more vulnerable to the COVID-19 crisis's effects, necessitating action to protect them [13]. During the COVID-19 pandemic, the prevalence of mental illnesses in pregnant women was significantly higher than before the pandemic [7, 14, 15, 16].

GAD is associated with a high rate of comorbidity with psychiatric and medical illnesses, as well as a significant degree of social handicap [17], with 27 percent of GAD patients reporting moderate or severe social disability [18, 19]. Anxiety and depression were the most common mental disorders among pregnant women [20, 21]. The risk factors of GAD among pregnant mothers were lower perceived support [22], being female, and younger age [23], lower educational status, low income or socioeconomic status [9, 23, 24, 25], pre-existing mental illness [26, 27], self-rated health status and social isolation [28], family history of mental health, and who experienced greater disruption in daily life [25], rural residence, previous complication, and multiparty pregnancy status [9, 24]. Furthermore, COVID-19 infection-related symptoms, contact with COVID-19-affected people, increased antenatal COVID-19 anxiety, and the level of information about COVID-19 have all been linked to a decline in mental health [29, 30, 31, 32].

There are no sufficient studies in poor nations on the psychological effect of the COVID-19 pandemic on mothers attending prenatal care. Although ordinal logistic regression is a widely used approach in clinical research, there is a paucity of literature on the ordered outcome of GAD. The proportional odds model (POM) is the most widely used logistic regression model for assessing ordinal response variables [33, 34]. Therefore, the current study aimed to determine the prevalence of generalized anxiety disorder symptoms during COVID-19, and its associated factor among mothers attending perinatal service in the study area during the pandemic through the application of ordinal logistics regression.

## 2 Material and methods

### 2.1 Study design and setting

The hospital-based cross-sectional study was conducted from July 10^th^, 2020 to August 10^th^, 2020 at Kembata Tembaro zone, in the Southern Ethiopia. The zone is located three-hundred six (306) kilometers far from Addis Ababa, which is the capital city of Ethiopia through Hosanna. There are four primary hospitals, and one general hospital, in the zone.

### 2.2 Sample size and sampling procedure

We have selected two primary hospitals, one general hospital out of four primary hospitals, and one general hospital in the Kembata Tembaro zone purposively. A single population proportion formula and a simple random sampling technique were used to calculate the sample size. The sample size needed for the study was calculated by assuming 50% of the prevalence of generalized anxiety disorder among mothers in a single population fraction for unknown prevalence, with a margin of error of 5%. Then the required sample size for current study becomes:

(1) $$n=\frac{{\left(1.96\right)}^{2}*\left(0.5\right)*\left(0.5\right)}{{\left(0.05\right)}^{2}}=384.5$$

**Figure 1:**
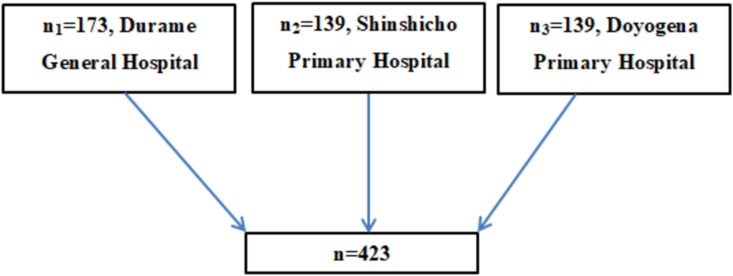
Sampling procedure of mothers attending each hospital.

By assuming a non-response rate of 10% the final sample becomes 384.5 + 38.45 = 422.95 ≈ 423. The number of mothers who visited each of the three hospitals is shown conceptual framework (Figure 1).

### 2.3 Measurement and Data Collection

Data was collected from mothers using a structured self-administered questionnaire. The measurement tools were created after reviewing various works of literature and the WHO guidelines. Sociodemographic characteristics, maternal health-related questions, medical history, alcohol use, psychosocial questions, and COVID-19-related questions were all included in the survey questionnaire.

The scale of generalized anxiety disorder (GAD-7) was used to assess the level of anxiety among mothers. GAD-7 is the standard tool used in several previous studies [35, 36]. It's a seven-item questionnaire designed to screen patients for anxiety and assess the severity of their symptoms. Each of items are rated on 4-point Likert-scale on response categories, such as “not at all”, “several days”, “more than half of the days”, and “nearly every day”, respectively, on the symptoms in the previous two weeks. The total score of GAD-7 is ranged from zero to twenty-one, with higher scores indicating more severe anxiety-related functional impairments. These scores represent 0–4 (non-minimal level of anxiety), 5–9 (mild level of anxiety), 10–14 (moderate level of anxiety), and 15–21 (severe level of anxiety). The results of the reliability analysis revealed a high level of internal consistency between the items (Cronbach's alpha = 0.876).

The Oslo-3 item was used in the current study to assess perceived social support. It is a three-item questionnaire that has been used in several studies to assess social support [37, 38]. The sum score scale runs from 3 to 14, with three levels of support: poor support (3–8), moderate support (9–11), and strong support (12–14). Cronbach's alpha for Oslo-3 PSS was 0.891 after we checked internal consistency and reliability.

### 2.4 Data Quality Control

To ensure consistency in meaning, a questionnaire was translated into Amharic and then back to English. Data collectors and supervisors received extensive training in data collection techniques as well as the instrument's components. Health professionals were included in data collection to ensure data quality. A pretest was conducted prior to the start of the data collection. The information was collected using a standard tool developed by experts. Every day, the authors and supervisors double-checked the data for accuracy.

### 2.5 Study variables

The response variable for this study was generalized anxiety disorder. Let Yi be the response variable, and i be the $i^{th}$ category of the response variable, then it is shown as follows:

$$Yi=\left\{ \begin{array}{l} 0,\;Non/minimal\;Anxiety\;\\ 1,\;Mild\;Anxiety\;\\ 2,\;Moderate\;Anxiety\;\\ 3,\;Severe\;Anxiety\; \end{array}\right.$$

Explanatory variables which are considered in the current study were as follows:

**Socio-demographic variables:** age of mother (continuous), residence (rural, town), monthly income (≤2000, 2001–5000, ≥5001 ET B), education status (no formal education, primary school, secondary school, diploma/some certificates, bachelor and above), occupation status (unemployed, health worker, other), and perceived social support (poor, moderate, strong).

**Substance use:** alcohol habit of mothers (no, yes).

**Maternal health-related factors:** number of pregnancies (prim gravida, multigravida), parity of pregnancy (prim parity, multiparity), pregnancy status (wanted, unwanted).

**Clinical factors:** chronic illness (no, yes), family history of anxiety/mood disorder (no, yes).

**COVID-19 related factors:** fear of contracting covid-19 (no, yes), and time spent in focusing on covid-19 news (in hours) (<1, 1–2, >3 h).

## 3 Method of the data analysis

Frequency, percentages, and graphs were used to report the descriptive results of the study. To identify the factors associated with an ordinal form of GAD, an ordered logit model was used. Before fitting the multivariable POM model, the effect of collinearity was checked. In stepwise ordinal logistic regression, covariates significant at a 25% [39, 40] level in the univariable were used as a subset of covariates for multivariable analysis. The Brant test was used in our study to test the proportionality assumption [41]. Finally, the model's performance was assessed using the Hosmer goodness of fit test [42].

### 3.1 Statistical models

The proportional odds model (POM) is widely utilized in the epidemiological as well as the biological applications [43, 44]. If the proportionality condition is violated, the partial proportional odds model (PPOM) may be a better option to analyze ordinal response variable [45, 46].

### 3.2 Proportional odds model (POM)

The assumption of the POM ensures that the odds ratios for all categories are the same. When the log odds ratio across the cut points is the same, then the proportional odds (PO) assumption is met, and the POM is used [44]. As previously stated, generalized anxiety disorder (GAD) represented by Yi observations are classified into one of four groups (non/minimal, mild, moderate, or severe). Similarly, covariates (xi) refer to the vector of covariates with the dimension p (i=1,2....p), which contains the observation on all p independent variables. As a result, we may express the dependency of the response variable (Y) on explanatory variables xi as follows:

(2) $$P\left(Y\geq y_{j}|x\right)=\frac{1}{1+\exp\left(-\alpha_{j}-{x_{i}}^{\prime }\beta \right)},j=1,2,3$$

It can also be defined as

(3) $$\log\left[\frac{P\left(Y\geq y_{j}|x\right)}{1-P\left(Y\geq y_{j}|x\right)}\right]=\alpha +{x_{i}}^{\prime }\beta ,for,j=1,2,3$$

Where $P\left(Y\;\geq \;y_{j}\right)$ is denoted as the cumulative probability of an event $\left(Y\;\geq \;y_{j}\right);\;\alpha j\;$ is the respective constant term/intercepts; *β* is the vector of regression coefficients with the dimension of (p by 1) that corresponds to the xi covariates in the model [47, 48].

### 3.3 Ethical approval and consent for participation

Ethical clearance for current study was obtained from the Mizan-Tepi University, College of Natural and Computational Science. A formal letter was written to the Kembata Tembaro zone Health Bureau for permission and support, and the zone then wrote a letter to the respective health center. Each study participant provided informed verbal consent. The study was conducted according to Helsinki declaration. The data were collected anonymously, and personal information was secured.

## 4 Results

### 4.1 Descriptive summaries of socio-demographic characteristics

The average age of the study participants in this study was 31.09 (SD = 3.72) years old. Of all 423 women attending perinatal service at the selected hospitals, more than half, 254 (60.0%) were town residents, of which 68(26.8%), 106(41.7%), 53(20.9%), and 27 (10.6%) were non/minimal, mild, moderate, and severe anxiety respectively. A large percentage of study participants 242 (57.2%) had monthly income between 2001-5000 ET B, while only 37 (8.7%) had an income of more than 5001 ET B.

Regarding the education status of participants 64 (15.1%) had no formal education, 187 (44.2%) had a primary school, 93 (22.0%) had secondary school, 53 (12.5%) had diploma/some certificates, and 26 (6.1%) had bachelor and above. When looking at occupation status, a small percentage of 29 (6.9%) were health workers, of which 19(65.5%), 6(20.7%), 3(10.3%), and 1 (3.4%) had non/minimal to severe GAD respectively. On the other hand, of all mothers, 268 (63.4%) had occupations other than health work, and 126 (29.8%) were unemployed (Table 1).

**Table 1:** Sociodemographic characteristics of women attending perinatal service.

Variables	Categories	Total N (%)	Level of Generalized anxiety disorder			
			Non/minimal	Mild	Moderate	Severe
			N (%)	N (%)	N (%)	N (%)
Age	Continuous	Average age = 31.09 Standard deviation (SD) = 3.72				
Residence	Rural	169 (40.0)	66 (39.1)	65 (38.5)	32 (18.9)	6 (3.6)
	Town	254 (60.0)	68 (26.8)	106 (41.7)	53 (20.9)	27 (10.6)
Monthly income	≤2000 ET B	144 (34.0)	44 (30.6)	62 (43.1)	24 (16.7)	14 (9.7)
	2001-5000 ET B	242 (57.2)	77 (31.8)	92 (38.0)	57 (23.6)	16 (6.6)
	≥5001 ET B	37 (8.7)	13 (35.1)	17 (45.9)	4 (10.8)	3 (8.1)
Education status	No formal education	64 (15.1)	20 (31.3)	28 (43.8)	10 (15.6)	6 (9.4)
	Primary school	187 (44.2)	54 (28.9)	79 (42.2)	41 (21.9)	13 (7.0)
	Secondary school	93 (22.0)	23 (24.7)	37 (39.8)	20 (21.5)	13(v)
	Diploma or some certificates	53 (12.5)	24 (45.3)	17 (32.1)	11 (20.8)	1 (1.9)
	Bachelor and above	26 (6.1)	13 (50.0)	10 (38.5)	3 (11.5)	0 (0.0)
Occupation	Unemployed	126 (29.8)	32 (25.4)	52 (41.3)	23 (18.3)	19 (15.1)
	Health worker	29 (6.9)	19 (65.5)	6 (20.7)	3 (10.3)	1 (3.4)
	Other	268 (63.4)	83 (31.0)	113 (42.2)	59 (22.0)	13 (4.9)

Source: Self-survey 2020

## 5 Maternal health, substance use, and COVID-19 related factors

Of all 423 participants, more than two-thirds of study participants 337 (79.7%) had a multigravida number of pregnancies. Regarding parity of pregnancy more than three fourth, 359 (84.9%) had multiparity. In the current study, only 36 (8.5%) of study participants were alcohol users, of which 19 (52.8%) had severe anxiety symptoms. when we look at the time spent on focusing COVID-19 news, a relatively higher percentage of mothers spent 1–2 h per day 185 (43.7), followed by those spent below 1 h per day 163 (38.5%). Regarding perceived social support, 160(37.8%), 200(47.3%), and 63 (14.9%) of mothers had poor, moderate, and strong level social support respectively. Regarding pregnancy status, 35 (8.3%) had an unwanted pregnancy. About 38 (9.0%) of women had a chronic illness of which 12 (31.6%), and 22 (57.9%) had moderate, and severe anxiety respectively. About 161 (38.1%) responded as they had fear of contracting COVID-19. Of all women, 30 (7.1%) had a family history of anxiety/mood disorder, of which 20.0%, and 70.0% had moderate, and severe anxiety symptoms respectively (Table 2).

**Table 2:** Maternal health, Substance use, and COVID-19 related factors.

Variables	Categories	Total N (%)	Level of Generalized anxiety disorder			
			Non/minimal	Mild	Moderate	Severe
			N (%)	N (%)	N (%)	N (%)
Number of pregnancies	Prim gravida	86 (20.3)	28 (32.6)	35 (40.7)	20 (23.3)	3 (3.5)
	Multigravida	337 (79.7)	106 (31.5)	136 (40.4)	65 (19.3)	30 (8.9)
Parity of pregnancy	Prim parity	64 (15.1)	17 (26.6)	31 (48.4)	13 (20.3)	3 (4.7)
	Multiparity	359 (84.9)	117 (32.6)	140 (39.0)	72 (20.1)	30 (8.4)
Pregnancy status	Wanted	35 (8.3)	11 (31.4)	19 (54.3)	3 (8.6)	2 (5.7)
	Unwanted	388 (91.7)	123 (31.7)	152 (39.2)	82 (21.1)	31 (8.0)
Alcohol habit	No	387 (91.5)	133 (34.4)	165 (42.6)	75 (19.4)	14 (3.6)
	Yes	36 (8.5)	1 (2.8)	6 (16.7)	10 (27.8)	19 (52.8)
Time spent on COVID-19 news per day (in hours)	<1	163 (38.5)	53 (32.5)	66 (40.5)	28 (17.2)	16 (9.8)
	1–2	185 (43.7)	60 (32.4)	73 (39.5)	44 (23.8)	8 (4.3)
	>3	75 (17.7)	21 (28.0)	32 (42.7)	13 (17.3)	9 (12.0)
Perceived Social support	Poor	160 (37.8)	37 (23.1)	57 (35.6)	40 (25.0)	26 (16.3)
	Moderate	200 (47.3)	68 (34.0)	91 (45.5)	35 (17.5)	6 (3.0)
	Strong	63 (14.9)	29 (46.0)	23 (36.5)	10 (15.9)	1 (1.60)
Chronic illness	No	385 (91.0)	133 (34.5)	168 (43.6)	73 (19.0)	11 (2.9)
	Yes	38 (9.0)	1 (2.6)	3 (7.9)	12 (31.6)	22 (57.9)
Fear of contracting covid-19	No	262 (61.9)	104 (39.7)	96 (36.6)	45 (17.2)	17 (6.5)
	Yes	161 (38.1)	30 (18.6)	75 (46.6)	40 (24.8)	16 (9.9)
Family history of anxiety/mood disorder	No	393 (92.9)	134 (34.1)	168 (42.7)	79 (20.1)	12 (3.1)
	Yes	30 (7.1)	0 (0.0)	3 (10.0)	6 (20.0)	21 (70.0)

Source: Self-survey 2020

### 5.1 The prevalence of generalized anxiety disorder among women

**Figure 2:**
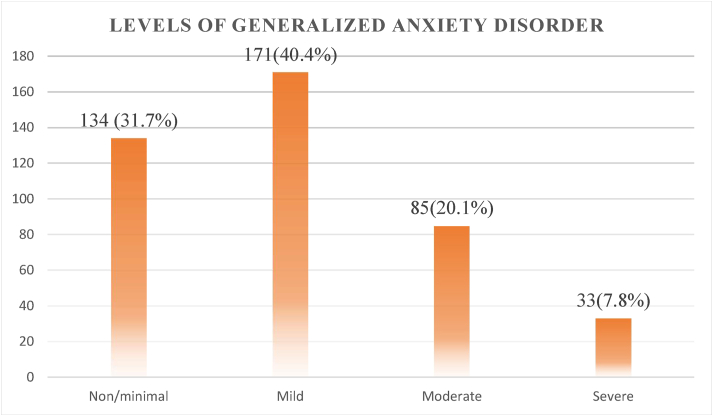
Levels of generalized anxiety disorder among mothers attending perinatal service.

The prevalence of generalized anxiety disorder (GAD) among women attending perinatal service during COVID-19 were 134 (31.7%), 171(40.4%), 85(20.1%), and 33 (7.8%) had minimal to severe generalized anxiety disorder respectively. From this, we can also observe that 118 (27.9%) mothers had moderate to severe generalized anxiety disorder (Figure 2).

### 5.2 Univariable analysis

From the univariable analysis the covariate age of women, residence, monthly income, occupation, education, alcohol habit, number of pregnancies, pregnancy status, chronic illness, family history, and fear of contracting COVID-19 were found to be significant at 25% level of significance, while parity of pregnancy and time spent on the COVID-19 related news per day were not significant at univariable level. As a result of this findings, we should remove the variables parity of pregnancy, & the time spent on the news related to the COVID-19 per day and shall do our multivariable proportional odds model (POM) analysis based on the remaining variables. Therefore, the impact of the covariate age of women, residence, monthly income, occupation, education status, alcohol habit, pregnancy status, number of pregnancies, chronic illness, family history, and fear of contracting COVID-19 women shall be analyzed using the multivariable POM (Table 3).

**Table 3:** Univariable and Multivariable Proportional odds model.

Variables	Categories	cPOR	aPOR	p-values	95%CI for aPOR
Age of women	Continuous	0.960∗	0.866	0.456	[0.593–1.264]
Residence (ref: Rural)	Town	1.736∗	1.827	0.003∗∗	[1.233–2.708]
Monthly income (ref: ≤2000 ET B)	2001-5000 ET B	0.479∗	0.351	0.229	[0.028–1.014]
	≥5001 ET B	0.417∗	0.374	0.422	[0.033–1.086]
Occupation (ref: unemployed)	Health worker	0.184∗	0.117	<0.001∗∗	[0.044–0.311]
	Other	0.687∗	0.509	0.011∗∗	[0.303–0.857]
Education status (ref: No formal education)	Primary school	0.921	0.824	0.088	[0.138–1.509]
	Secondary school	0.739	0.711	0.077	[0.253–1.168]
	Diploma or some certificates	0.617∗	0.978	0.954	[0.463–2.065]
	Bachelor and above	0.438∗	0.650	0.367	[0.255–1.657]
Alcohol (ref: No)	Yes	1.319∗	3.437	0.007∗∗	[1.397–8.454]
Parity (ref: Prim parity)	Multi parity	0.961	-	-	-
Pregnancy status (Wanted)	Unwanted	1.180∗	1.145	0.218	[0.866–1.291]
No of pregnancy (ref: Prim gravida)	Multi gravida	1.140∗	1.162	0.529	[0.728–1.855]
Chronic illness (ref: No)	Yes	40.53∗	7.685	<0.001∗∗	[3.045–19.39]
Family history (ref: No)	Yes	61.56∗	7.839	<0.001∗∗	[2.656–23.12]
Social support (ref: poor)	Moderate	0.428∗	0.648	0.044∗∗	[0.425–0.989]
	Strong	0.288∗	0.495	0.021∗∗	[0.272–0.901]
Time spent on COVID-19 news per day (ref: <1 h)	1–2 h	0.975	-	-	-
	>3 h	1.196	-	-	-
Fear of COVID-19 (ref: No)	Yes	2.155∗	1.704	0.008∗∗	[1.152–2.521]

Parallel line test: p-value = 0.392, Goodness of fit test of overall model: Deviance (Chi-Square =638.582, df = 927, p-value = 1.000), Nagelkerke's R^2^ = 0.498

∗Significant at 25%, ∗∗ significance at 5%, cPOR: crude proportional odds ratio, aPOR: Adjusted proportional odds ratio.

### 5.3 Multivariable proportional odds model

The covariates in the multivariable POM were chosen based on the unvariable association at a 25% level of significance. We checked for collinearity before developing the multivariable ordinal logistic regression model, and it was not present in the current dataset. The overall assumption of proportionality in this study was not violated, since p-value = 0.392 which exceeds 0.05. The main assumption of the ordered logit model is the assumption of proportional odds (PO), which stated that each independent variable in the model has an identical effect at each cumulative split of the ordinal dependent variable. This assumption is held in the current study. As a result, the proportional odds model was fitted for the data set (Table 3).

### 5.4 Factors associated with generalized anxiety disorder among mothers

According to results of multivariable POM, variables such as residence, monthly income, occupation, education, chronic illness, family history, and fear of COVID-19 were significantly related to a generalized anxiety disorder among pregnant mothers attending perinatal service at a 5% level of significance.

The findings showed that the risk of having a higher order of GAD was nearly twice, 1.827 [aPOR = 1.827; 95% CI: 1.233–2.708] times higher among town residents as compared to rural residents. On the other hand, mothers who had any occupation other than health worker were 0.509 [aPOR = 0.509, 95%CI: 0.303–0.857] times less likely to develop GAD as compared to unemployed mothers. Moreover, a woman whose occupation is health care workers were 0.117 [aPOR = 0.117, 95% CI = 0.044–0.311] times less likely to experience the higher level of anxiety than those who were unemployed. This might be because this group of mothers has a better understanding of pandemic and its prevention measures as compared to other populations.

Mothers who had alcohol habits were 3.437 [aPOR = 3.437, 95% CI = 1.397–8.454] times more likely to develop a higher level of anxiety disorder as compared to those who had no alcohol habit. Likewise, those who had a chronic illness were 7.685 times [aPOR = 7.685, 95% CI = 3.045–19.39] more likely to experience the higher level of anxiety as compared to mothers who had no chronic illness. Similarly, mothers having a family history of anxiety had 7.839 times [aPOR = 7.839, 95% CI = 2.656–23.12] higher odds of anxiety as compared to their counterparts.

Among pregnant mothers, social support was found to be statistically associated with generalized anxiety disorder. Those who had moderate social support were 0.648 [aPOR = 0648, 95% CI = 0.425–0.989] times less likely to develop the generalized anxiety disorder as compared to those who had poor social support. Similarly, the odds of mothers who had strong social support were found to be 0.495 [aPOR = 0.495, 95% CI = 0.272–0.901] times lower as compared to mothers who had poor social support to develop a generalized anxiety disorder.

Fear of COVID-19 was found to be a statistically significant predictor of generalized anxiety among pregnant mothers attending perinatal service. Those mothers who had fear of contracting COVID-19 were 1.704 [aPOR = 1.704, 95% CI = 1.152–2.521] times higher odds to develop a higher level of an anxiety disorder (Table 3).

### 5.5 Adequacy of the fitted model

The overall model's goodness of fit test revealed that the deviance statistic (Chi-Square = 638.582, df = 927, p-value = 1.000) had a large p-value. As a result, we do not reject the null hypothesis and conclude that the model adequately fits the data. In addition to that the Nagelkerke's R^2^ = 0.498 indicated that independent variables in the model explained 49.8 percent of the variation among GAD (Table 3).

## 6 Discussion

The main purpose of the current study was to use an Ordered logit model to assess determinants of GAD among mothers attending perinatal services at three selected hospitals during COVID-19 lockdown. To apply ordinal logistic regression, the study assumed the ordered nature of GAD categories as non-minimal, mild, moderate, and severe anxiety disorder based on GAD-7 standard cut point.

The current study found that 134 (31.7%), 171 (40.4%), 85 (20.1%), and 33 (7.8%) of women attending perinatal services during COVID-19 had non/minimal to severe GAD, respectively. The overall prevalence of GAD symptoms (moderate/severe) in our study was found to be 27.9%. This is consistent with the report of a prior study conducted among mothers attending a perinatal service of DURH, Ethiopia [49]. A study from China reported that the prevalence of GAD among pregnant mothers was 17.2%, which is lower than the current study's result [23]. It could be attributed to differences in the study design used, culture, facility, as well as the assessment tool implemented in both studies. Another study conducted in the United States during the COVID-19 Pandemic found that 22.7% of perinatal women had generalized anxiety [27]. Furthermore, a similar study from Qatar, reported that 34.4% of mothers who attend perinatal service had anxiety issues [50]. The disparity could be explained by differences in population, culture, and healthcare facilities.

However, the study before the outbreak of the pandemic reported that the prevalence of perinatal GAD was 8.5%–10.5% during pregnancy and 4.4%–10.8% postpartum [51]. Another study conducted prior to the COVID-19 outbreak also found a lower prevalence of GAD, 23.7% [52] than the current study. This disparity could be attributed to the impact COVID-19 pandemic on mental health. Although anxiety affects the entire population, perinatal women have reported significantly more anxiety during the pandemic. Concerns about COVID [53]. Pregnant women expressed concerns about their birth, and the COVID-19 outbreak did increase pregnant women's levels of anxiety that are both general and health anxiety levels [54]. According to Green et al., pre-pandemic anxiety in perinatal women was one in five, but has since increased with the onset of the COVID-19 pandemic [53].

In the proportional odds model (POM), variables such as residence, monthly income, occupation, chronic illness, family history, stressful life events, and fear of COVID-19 were statistically significantly related with a GAD.

The current study finding showed that the probability of being in the higher order of anxiety among town residents was 1.527 times more likely to develop a general anxiety disorder as compared to those from rural areas. This is in agreement with a previous study that rural residents are less likely to develop GAD [49]. Another study conducted among pregnant women referred to health centers in Zanjan province, Iran, at the second peak of COVID-19 found similar results [24]. Individuals in rural areas are more dispersed and have less opportunity of coming into touch with strangers and other people, resulting in a lower risk of infection. This group of people has less concern and worry about becoming infected than those who live in cities.

Participants who had higher monthly income (more than 5001 ET B) were less likely to develop higher-order anxiety as compared to lower-income participants. This is supported by a previous study that the poorest had higher rates of GAD [55], also it replicates the findings of a Singaporean study [56]. Another study found that individuals who were concerned about work delays and lost income were directly influenced by the unclear job resumption time, indicating that stressful income situations can exacerbate common mental health issues such as anxiety, depression, and others [57]. Furthermore, numerous prior studies have revealed that lower-income and socioeconomic levels have a substantial impact on anxiety disorders [9, 23, 24, 25].

On the other hand, mothers whose occupation is health care workers had 0.325-times lower risk to experiencing a sever (higher order) of anxiety than those who were unemployed. This might be attributed to health workers having a better knowledge of self-protection of the pandemic as compared to ordinary populations. A prior study reported that GAD was correlated with unemployment [17].

Our study results revealed that mothers who had a chronic illness were 3.043 times more likely to experience a higher level of anxiety disorder as compared to those who had no chronic illness. This is supported by a previous study [17], which reported that peoples with chronic illness have a higher likelihood of experiencing anxiety disorder than others. Moreover, another study reported that chronic illness had a significant effect on comorbid anxiety disorder [58]. Literature supported that having a chronic illness, as well as misusing addictive substances, can increase the likelihood of developing an anxiety disorder. Having a chronic health condition can also lead to anxiety [59].

Similarly, mothers with a positive family history of the anxiety disorder were more diagnosed with GAD than those with a negative family history. Moreover, study reported that GAD was more diagnosed among patients with positive family history than the negative family history [25, 52]. According to report of different studies, first-degree relatives of someone with GAD are more likely to develop mood and anxiety disorders in general, with an increased risk of developing GAD in particular [60, 61]. Moreover, previous evidence indicates that people with pre-pandemic mental disorders are more vulnerable to COVID-19-related stress than the general public [62, 63], & this might be as a result of poorer coping abilities, disruptions in mental health care routines, and increased risk of relapse or exacerbation of symptoms [64].

Perceived support was found to be a risk factor for GAD among pregnant mothers who attend perinatal services. This is consistent with a previous study that reported that lower perceived support has a substantial link with generalized anxiety disorder [22]. Having a moderate or higher level of social support has been shown to reduce the risk of affective disorders by mitigating the effects of stress and improving coping strategies. The previous study has also found that social support is an important protective factor against anxiety and depression [65, 66, 67, 68], and It may be especially important to improve psychological well-being and prevent mental disorders during times of crisis, such as the COVID-19 pandemic [69].

Fear of COVID-19 was found to be a statistically significant predictor of generalized anxiety among perinatal women. Those women who fear COVID-19 were 2.178 times higher odds to develop a higher level of anxiety disorder as compared to their counterparts. A similar study reported that fear and uncertainty related to the spread of the virus were one of the most important predictors of emotional distress [70]. Concerns about the risk of infection with COVID19 have also contributed to the rise in anxiety disorders [71]. Fear of contracting the disease or of the virus's potential effects on the fetus or newborn may cause increased anxiety in the pregnant mother [72].

### 6.1 Limitations and strengths of the study

This study tried to assess generalized anxiety disorder among mothers attending perinatal service. Some of the limitations while conducting the current study were, first, we cannot prove a causal relationship in this cross-sectional study. As a second point, due to limited resources and the urgency of the COVID-19 outbreak, might influence the data collection process, and response. Despite this limitation, one of the study's strengths was that, because the response variable GAD has an ordinal nature, we used ordinal logistics regression rather than imposing its categories with natural order into a binary category. In addition to this, the current study is one of the few in poor nations that employ standardized methods and conduct rigorous analyses.

### 6.2 Directions for future research

The Generalized Anxiety Disorder 7 (GAD-7) assessment tool was used in this study to screen perinatal mothers for generalized anxiety disorder. We suggest using different assessment tools for GAD in future research, like Generalized Anxiety Disorder Severity Scale (GADSS) [73], Generalized Anxiety Disorder Questionnaire-Iv (GADQ-IV) [74], Leibowitz Social Anxiety Scale (LSAS) [75], Overall Anxiety Severity And Impairment Scale (OASIS) [76, 77], Hospital Anxiety And Depression Scale (HADS) [78] for further assessment. The current study did not investigate differences in the hospital delivering the service, care for pandemic protection, and interventions, despite the fact that this may be an important factor to consider because pandemic concerns can lead to mental health issues [79]. Generalized anxiety disorder (GAD) is very common in the general population and it is also the most common anxiety disorder seen in primary care [80]. Therefore, future research should look into whether GAD is more prevalent in pregnant and postpartum women than in the general population, and also GAD would be compared before and after vaccination of COVID-19. In addition, a larger investigation is required to see the link between the COVID-19 pandemic and psychological health issues in other parts of the country in broader scope, as well as their long-term consequences.

## 7 Conclusions

Current study findings showed that the magnitude of generalized anxiety disorder among mothers attending the perinatal service was high. The covariates like being town residents, lower-income status, occupation status, having a chronic illness, having a positive family history of anxiety or mood disorder, and fear of the COVID-19 were contributing to has a significant impact on the GAD among mothers. Mothers attending perinatal services should be given special attention, as well as they should be taught how to cope with the psychological impact of COVID-19 in order to receive better health care.

## Declarations

### Author contribution statement

Mesfin Esayas Lelisho: Conceived and designed the experiments; Performed the experiments; Analyzed and interpreted the data; Contributed reagents, materials, analysis tools or data; Wrote the paper. Amanuel Mengistu Merera; Seid Ali Tareke; Sali Suleman Hassen; Sebwedin Surur Jemal; Admasu *Markos kontuab*; Meseret Mesfin Bambo: Contributed reagents, materials, analysis tools or data; Wrote the paper.

### Funding statement

This research did not receive any specific grant from funding agencies in the public, commercial, or not-for-profit sectors.

### Data availability statement

Data will be made available on request.

### Declaration of interests statement

The authors declare no conflict of interest.

### Additional information

No additional information is available for this paper.

## Appendix A Supplementary data

GAD Questionnaire_01 GAD Questionnaire_01

## References

[1] Baldwin D., Stein M.B., Hermann R. (2018).

[2] Akiskal H.S. (1998). Toward a definition of generalized anxiety disorder as an anxious temperament type. Acta Psychiatr. Scand..

[3] Crocq M.-A. (2017). The history of generalized anxiety disorder as a diagnostic category. Dialogues Clin. Neurosci..

[4] Robertson E., Grace S., Wallington T., Stewart D.E. (2004). Antenatal risk factors for postpartum depression: a synthesis of recent literature. Gen. Hosp. Psychiatr..

[5] Zhong Q.-Y., Gelaye B., Zaslavsky A.M., Fann J.R., Rondon M.B., Sánchez S.E., Williams M.A. (2015). Diagnostic validity of the generalized anxiety disorder-7 (GAD-7) among pregnant women. PLoS One.

[6] Wu Y., Zhang C., Liu H., Duan C., Li C., Fan J., Li H., Chen L., Xu H., Li X. (2020). Perinatal depressive and anxiety symptoms of pregnant women during the coronavirus disease 2019 outbreak in China. Am. J. Obstet. Gynecol..

[7] Hessami K., Romanelli C., Chiurazzi M., Cozzolino M. (2020). COVID-19 pandemic and maternal mental health: a systematic review and meta-analysis. J. Matern. Neonatal Med..

[8] Cameron E.E., Joyce K.M., Delaquis C.P., Reynolds K., Protudjer J.L.P., Roos L.E. (2020). Maternal psychological distress & mental health service use during the COVID-19 pandemic. J. Affect. Disord..

[9] Pandey D. (2020). The prevalence of general anxiety disorder and its associated factors among women’s attending at the perinatal service of Dilla University referral hospital, Dilla town, Ethiopia, April, 2020 in Covid pandemic. Heliyon.

[10] Staneva A., Bogossian F., Pritchard M., Wittkowski A. (2015). The effects of maternal depression, anxiety, and perceived stress during pregnancy on preterm birth: a systematic review. Women Birth.

[11] Schetter C.D., Tanner L. (2012). Anxiety, depression and stress in pregnancy: implications for mothers, children, research, and practice. Curr. Opin. Psychiatr..

[12] Tribe R.M., Taylor P.D., Kelly N.M., Rees D., Sandall J., Kennedy H.P. (2018). Parturition and the perinatal period: can mode of delivery impact on the future health of the neonate?. J. Physiol..

[13] Janik K., Cwalina U., Iwanowicz-Palus G., Cybulski M. (2021). An assessment of the level of COVID-19 anxiety among pregnant women in Poland: a cross-sectional study. J. Clin. Med..

[14] Koyucu R.G., Karaca P.P. (2021). The covid 19 outbreak: maternal mental health and associated factors. Midwifery.

[15] López-Morales H., Del Valle M.V., Canet-Juric L., Andrés M.L., Galli J.I., Poó F., Urquijo S. (2021). Mental health of pregnant women during the COVID-19 pandemic: a longitudinal study. Psychiatr. Res..

[16] Howland M.A., Kotlar B., Davis L., Shlafer R.J. (2021). Depressive symptoms among pregnant and postpartum women in prison. J. Midwifery Wom. Health.

[17] Wittchen H. (2002). Generalized anxiety disorder: prevalence, burden, and cost to society. Depress. Anxiety.

[18] Lieb R., Becker E., Altamura C. (2005). The epidemiology of generalized anxiety disorder in Europe. Eur. Neuropsychopharmacol.

[19] Hynninen K.M.J., Breitve M.H., Wiborg A.B., Pallesen S., Nordhus I.H. (2005). Psychological characteristics of patients with chronic obstructive pulmonary disease: a review. J. Psychosom. Res..

[20] Luong-Thanh B.-Y., Nguyen L.H., Murray L., Eisner M., Valdebenito S., Hoang T.D., Phuc Do H., Van Vo T. (2021). Depression and its associated factors among pregnant women in central Vietnam. Heal. Psychol. Open..

[21] Ahorsu D.K., Imani V., Lin C.-Y., Timpka T., Broström A., Updegraff J.A., Årestedt K., Griffiths M.D., Pakpour A.H. (2020). Associations between fear of COVID-19, mental health, and preventive behaviours across pregnant women and husbands: an actor-partner interdependence modelling. Int. J. Ment. Health Addiction.

[22] Tikka S.K., Parial S., Pattojoshi A., Bagadia A., Prakash C., Lahiri D., Jaiswal J., Puri M., Kukreti P., Behera R.N. (2021). Anxiety among pregnant women during the COVID-19 pandemic in India− A multicentric study. Asian J. Psychiatr..

[23] Liu X., Chen M., Wang Y., Sun L., Zhang J., Shi Y., Wang J., Zhang H., Sun G., Baker P.N. (2020). Prenatal anxiety and obstetric decisions among pregnant women in Wuhan and Chongqing during the COVID-19 outbreak: a cross-sectional study. BJOG An Int. J. Obstet. Gynaecol..

[24] Maleki A., Ashtari M., Molaie P., Youseflu S. (2021). Influential factors of general anxiety disorder among Iranian pregnant women during the second peak of COVID-19 pandemic. Psychol. Health Med..

[25] McDonald H.M., Sherman K.A., Kasparian N.A. (2021). Factors associated with psychological distress among Australian women during pregnancy. Pers. Indiv. Differ..

[26] Wenzel A., Haugen E.N., Jackson L.C., Robinson K. (2003). Prevalence of generalized anxiety at eight weeks postpartum. Arch. Womens. Ment. Health.

[27] Liu C.H., Erdei C., Mittal L. (2021). Risk factors for depression, anxiety, and PTSD symptoms in perinatal women during the COVID-19 Pandemic. Psychiatr. Res..

[28] Xiong J., Lipsitz O., Nasri F., Lui L.M.W., Gill H., Phan L., Chen-Li D., Iacobucci M., Ho R., Majeed A. (2020). Impact of COVID-19 pandemic on mental health in the general population: a systematic review. J. Affect. Disord..

[29] Al Zubayer A., Rahman M.E., Islam M.B., Babu S.Z.D., Rahman Q.M., Bhuiyan M.R.A.M., Khan M.K.A., Chowdhury M.A.U., Hossain L., Bin Habib R. (2020). Psychological states of Bangladeshi people four months after the COVID-19 pandemic: an online survey. Heliyon.

[30] Nowacka U., Kozlowski S., Januszewski M., Sierdzinski J., Jakimiuk A., Issat T. (2021). COVID-19 pandemic-related anxiety in pregnant women. Int. J. Environ. Res. Publ. Health.

[31] Zeng X., Li W., Sun H., Luo X., Garg S., Liu T., Zhang J., Zhang Y. (2020). Mental health outcomes in perinatal women during the remission phase of COVID-19 in China. Front. Psychiatr..

[32] Spinola O., Liotti M., Speranza A.M., Tambelli R. (2020). Effects of COVID-19 epidemic lockdown on postpartum depressive symptoms in a sample of Italian mothers. Front. Psychiatr..

[33] Bender R., Grouven U. (1998). Using binary logistic regression models for ordinal data with non-proportional odds. J. Clin. Epidemiol..

[34] Fullerton A.S., Xu J. (2012). The proportional odds with partial proportionality constraints model for ordinal response variables. Soc. Sci. Res..

[35] Soto Balbuena M.C., de la F. Rodríguez Muñoz M., Le H.-N. (2021).

[36] Spitzer R.L., Kroenke K., Williams J.B.W., Löwe B. (2006). A brief measure for assessing generalized anxiety disorder: the GAD-7. Arch. Intern. Med..

[37] Kocalevent R.-D., Berg L., Beutel M.E., Hinz A., Zenger M., Härter M., Nater U., Brähler E. (2018). Social support in the general population: standardization of the Oslo social support scale (OSSS-3). BMC Psychol.

[38] Dalgard O.S., Dowrick C., Lehtinen V., Vazquez-Barquero J.L., Casey P., Wilkinson G., Ayuso-Mateos J.L., Page H., Dunn G. (2006). Negative life events, social support and gender difference in depression. Soc. Psychiatr. Psychiatr. Epidemiol..

[39] Bursac Z., Gauss C.H., Williams D.K., Hosmer D.W. (2008). Purposeful selection of variables in logistic regression. Source Code Biol. Med..

[40] Hosmer D.W., Lemeshow S., Sturdivant R.X. (2013).

[41] Brant R. (1990).

[42] Hosmer D.W. (2000). Assessing the fit of the model. Appl. Logist. Regres..

[43] Cox C., Chuang C. (1984). A comparison of chi-square partitioning and two logit analyses of ordinal pain data from a pharmaceutical study. Stat. Med..

[44] McCullagh P. (1980). Regression models for ordinal data. J. R. Stat. Soc. Ser. B..

[45] Long J.S., Freese J. (2006).

[46] V Ananth C., Kleinbaum D.G. (1997). Regression models for ordinal responses: a review of methods and applications. Int. J. Epidemiol..

[47] Lelisho M.E., Wogi A.A., Tareke S.A. (2022). Ordinal logistic regression analysis in determining factors associated with socioeconomic status of household in tepi town, southwest Ethiopia. Sci. World J..

[48] Das S., Rahman R.M. (2011). Application of ordinal logistic regression analysis in determining risk factors of child malnutrition in Bangladesh. Nutr. J..

[49] Kassaw C., Pandey D. (2021). COVID-19 pandemic related to anxiety disorder among communities using public transport at Addis Ababa, Ethiopia, march 2020: cross-sectional study design. Hum. Arenas.

[50] Farrell T., Reagu S., Mohan S., Elmidany R., Qaddoura F., Ahmed E.E., Corbett G., Lindow S., Abuyaqoub S.M., Alabdulla M.A. (2020). The impact of the COVID-19 pandemic on the perinatal mental health of women. J. Perinat. Med..

[51] Misri S., Abizadeh J., Sanders S., Swift E. (2015). Perinatal generalized anxiety disorder: assessment and treatment. J. Wom. Health.

[52] Barghouti F.F., Masalha A.I., Fayyomi H., Mari’e L.O., Ahmad M.M. (2018). Prevalence of generalized anxiety disorder in family practice clinics. Clin. Pract..

[53] Green S.M., Inness B., Furtado M., McCabe R.E., Frey B.N. (2022). Evaluation of an augmented cognitive behavioural group therapy for perinatal generalized anxiety disorder (GAD) during the COVID-19 pandemic. J. Clin. Med..

[54] Rathbone A.L., Cross D., Prescott J. (2022). Digit. Innov. Ment. Heal. Support.

[55] Ma X., Xiang Y., Cai Z., Lu J., Li S., Xiang Y., Guo H., Hou Y., Li Z., Li Z. (2009). Generalized anxiety disorder in China: prevalence, sociodemographic correlates, comorbidity, and suicide attempts. Psychiatr. Care.

[56] Lim L., Ng T.P., Chua H.C., Chiam P.C., Won V., Lee T., Fones C., Kua E.H. (2005). Generalised anxiety disorder in Singapore: prevalence, co-morbidity and risk factors in a multi-ethnic population, Soc. Psychiatry Psychiatr. Epidemiology.

[57] Chen J., Liu X., Wang D., Jin Y., He M., Ma Y., Zhao X., Song S., Zhang L., Xiang X. (2021). Risk factors for depression and anxiety in healthcare workers deployed during the COVID-19 outbreak in China, Soc. Psychiatry Psychiatr. Epidemiology.

[58] Bante A., Mersha A., Zerdo Z., Wassihun B., Yeheyis T. (2021). Comorbid anxiety and depression: prevalence and associated factors among pregnant women in Arba Minch zuria district, Gamo zone, southern Ethiopia. PLoS One.

[59] Culpepper L. (2009). Generalized anxiety disorder and medical illness. J. Clin. Psychiatr..

[60] Cassidy J., Lichtenstein-Phelps J., Sibrava N.J., Thomas C.L., Borkovec T.D. (2009). Generalized anxiety disorder: connections with self-reported attachment. Behav. Ther..

[61] Kaufman J., Charney D. (2000). Comorbidity of mood and anxiety disorders. Depress. Anxiety.

[62] Pan K.-Y., Kok A.A.L., Eikelenboom M., Horsfall M., Jörg F., Luteijn R.A., Rhebergen D., van Oppen P., Giltay E.J., Penninx B.W.J.H. (2021). The mental health impact of the COVID-19 pandemic on people with and without depressive, anxiety, or obsessive-compulsive disorders: a longitudinal study of three Dutch case-control cohorts. Lancet Psychiatr..

[63] Taylor S., Landry C.A., Paluszek M.M., Fergus T.A., McKay D., Asmundson G.J.G. (2020). COVID stress syndrome: concept, structure, and correlates. Depress. Anxiety.

[64] Druss B.G. (2020). Addressing the COVID-19 pandemic in populations with serious mental illness. JAMA Psychiatr..

[65] Labrague L.J., De los Santos J.A.A. (2020). COVID-19 anxiety among front-line nurses: predictive role of organisational support, personal resilience and social support. J. Nurs. Manag..

[66] Gariepy G., Honkaniemi H., Quesnel-Vallee A. (2016). Social support and protection from depression: systematic review of current findings in Western countries. Br. J. Psychiatry.

[67] Lelisho M.E., Tareke S.A. (2022). Prevalence and associated factors of depressive symptoms among mizan-tepi university students during the COVID-19 pandemic. J. Racial Ethn. Heal. Disparities.

[68] Tareke S.A., Lelisho M.E., Hassen S.S., Seid A.A., Jemal S.S., Teshale B.M., Wotale T.W., Pandey B.K. (2022). The prevalence and predictors of depressive, anxiety, and stress symptoms among tepi town residents during the COVID-19 pandemic lockdown in Ethiopia. J. Racial Ethn. Heal. Disparities.

[69] Saltzman L.Y., Hansel T.C., Bordnick P.S. (2020). Loneliness, isolation, and social support factors in post-COVID-19 mental health. Psychol. Trauma Theory, Res. Pract. Policy..

[70] Gambin M., Sękowski M., Woźniak-Prus M., Wnuk A., Oleksy T., Cudo A., Hansen K., Huflejt-Łukasik M., Kubicka K., Łyś A.E. (2021). Generalized anxiety and depressive symptoms in various age groups during the COVID-19 lockdown in Poland. Specific predictors and differences in symptoms severity. Compr. Psychiatr..

[71] Dorman-Ilan S., Hertz-Palmor N., Brand-Gothelf A., Hasson-Ohayon I., Matalon N., Gross R., Chen W., Abramovich A., Afek A., Ziv A. (2020). Anxiety and depression symptoms in COVID-19 isolated patients and in their relatives. Front. Psychiatr..

[72] Chen H., Selix N., Nosek M. (2021). Perinatal anxiety and depression during COVID-19. J. Nurse Pract..

[73] Shear K., Belnap B.H., Mazumdar S., Houck P., Rollman B.L. (2006). Generalized anxiety disorder severity scale (GADSS): a preliminary validation study. Depress. Anxiety.

[74] Newman M.G., Zuellig A.R., Kachin K.E., Constantino M. (2003). Generalized anxiety disorder questionnaire-IV, 8 fragebögen und ratingskalen zur. Gen. Angststörung..

[75] Rytwinski N.K., Fresco D.M., Heimberg R.G., Coles M.E., Liebowitz M.R., Cissell S., Stein M.B., Hofmann S.G. (2009). Screening for social anxiety disorder with the self-report version of the Liebowitz Social Anxiety Scale. Depress. Anxiety.

[76] Campbell-Sills L., Norman S.B., Craske M.G., Sullivan G., Lang A.J., Chavira D.A., Bystritsky A., Sherbourne C., Roy-Byrne P., Stein M.B. (2009). Validation of a brief measure of anxiety-related severity and impairment: the overall anxiety severity and impairment scale (OASIS). J. Affect. Disord..

[77] Hermans M., Korrelboom K., Visser S. (2015). A Dutch version of the overall anxiety severity and impairment scale (OASIS): psychometric properties and validation. J. Affect. Disord..

[78] Gallagher A., Kring D., Whitley T. (2020). Effects of yoga on anxiety and depression for high risk mothers on hospital bedrest, Complement. Ther. Clin. Pract..

[79] Talevi D., Socci V., Carai M., Carnaghi G., Faleri S., Trebbi E., di Bernardo A., Capelli F., Pacitti F. (2020). Mental health outcomes of the CoViD-19 pandemic. Riv. Psichiatr..

[80] Sapra A., Bhandari P., Sharma S., Chanpura T., Lopp L. (2020). Using generalized anxiety disorder-2 (GAD-2) and GAD-7 in a primary care setting. Cureus.

