# Corrigendum to “Generalized anxiety disorder among mothers attending perinatal services during COVID-19 pandemic: Using ordinal logistic regression model” [Heliyon Volume 8, Issue 6, June 2022, Article e09778]

**DOI:** 10.1016/j.heliyon.2025.143231

**Published:** 2025-03-17

**Authors:** Mesfin Esayas Lelisho, Amanuel Mengistu Merera, Seid Ali Tareke, Sali Suleman Hassen, Sebwedin Surur Jemal, Admasu Markos Kontuab, Meseret Mesfin Bambo

**Affiliations:** Department of Statistics, College of Natural and Computational Science, Mizan-Tepi University, P.O. Box 121, Tepi, Ethiopia

In the original published version of this article, ethics approval details associated with the study was not provided, which is not added to the revised version.

The original manuscript showed the statement as below:

Ethical clearance for current study was obtained from the Mizan-Tepi University, College of Natural and Computational Science. A formal letter was written to the Kembata Tembaro zone Health Bureau for permission and support, and the zone then wrote a letter to the respective health center. Each study participant provided informed verbal consent. The study was conducted according to Helsinki declaration. The data were collected anonymously, and personal information was secured.

The missing information has now been added in the correct version as below:

Ethical clearance for current study was approved by the Research and Ethical Review Board (RERB) of the College of Natural and Computational Science at Mizan-Tepi University on May 20, 2020, under ethics approval number RCS/161/09/12. A formal letter was written to the Kembata Tembaro zone Health Bureau for permission and support, and the zone then wrote a letter to the respective health center. Each study participant provided informed verbal consent. Due to the significant challenges posed by the COVID-19 pandemic in obtaining written consent, the Research Review Committee of the College of Natural and Computational Science at Mizan-Tepi University have waived the requirement for written informed consent. The study was conducted according to Helsinki declaration. The data were collected anonymously, and personal information was secured.

The authors apologize for the omission. Both the HTML and PDF versions of the article have been updated to correct the errors.

## Declaration of competing interest

The authors declare no conflict of interest.

