# Supplementary material for: Generalized anxiety disorder among mothers attending perinatal services during COVID-19 pandemic: using ordinal logistic regression model
Source: Heliyon. 2022 Jun 22;8(6):e09778. doi: 10.1016/j.heliyon.2022.e09778 (PMC9220756; doi:10.1016/j.heliyon.2022.e09778)
Supplement: GAD Questionnaire_01 [file mmc1.docx]

First and foremost, we would like to express our gratitude in advance for your cooperation and time commitment in providing this information. The goal of this questionnaire is to collect information that will be used to identify factors associated with generalized anxiety disorder among mothers attending perinatal services in the study area during COVID-19. All of the information you provided will be critical to the success of this study. The questionnaire is divided into three sections: sociodemographic characteristics of women attending perinatal services, maternal health, substance use, and COVID-19 related factors, and questions about the GAD-7 Anxiety Severity Score. Please answer them truthfully. Thank you so much for your assistance.

***Sociodemographic characteristics of women attending perinatal service***

1. Age __________________
2. Residence
3. Rural
4. Urban
5. Monthly income
6. ≤2000 ETB
7. 2001-5000 ETB
8. ≥5001 ETB
9. Education status
10. No formal education
11. Primary school
12. Secondary school
13. Diploma or some certificates
14. Bachelor and above
15. Occupation
16. Unemployed
17. Health worker
18. Other

***Maternal health, Substance use, and COVID-19 related factors***

1. Number of pregnancies
2. Prim gravida
3. Multigravida
4. Parity of pregnancy
5. Prim parity
6. Multiparity
7. Pregnancy status
8. Wanted
9. Unwanted
10. Do you have an alcohol habit?
11. No
    1. Yes
12. Time spent on COVID-19 news per day (in hours)
13. <1
14. 1-2
15. >3
16. Do you have any chronic illnesses?
17. No
18. Yes
19. Do you feel fear of contracting covid-19?
20. No
21. Yes
22. Do you have a family history of anxiety/mood disorder?
23. No
24. Yes
25. **Perceived Social support**

Oslo 1: How many people are so close to you that you can count on them if you have great personal problems?

1 ‘none’

2 ‘1–2’

3 ‘3–5’

4 ‘5+’

Oslo 2: How much interest and concern do people show in what you do?

1 ‘none’

2 ‘little’

3 ‘uncertain’

4 ‘some’

5 ‘a lot’

Oslo 3: How easy is it to get practical help from neighbors if you should need it?

1 ‘very difficult’

2 ‘difficult’

3 ‘possible’

4 ‘easy’

5 ‘very easy’

***Scoring GAD-7 Anxiety Severity***

In the following section, please respond to the following questions by placing a checkmark (√) in the answer box that corresponds to your response.

| Over the last two weeks, how often have you been bothered by the following problems? | Not at all | Several days | More than half the days | Nearly every day |
| --- | --- | --- | --- | --- |
| 1. Feeling nervous, anxious, or on edge |  |  |  |  |
| 2. Not being able to stop or control worrying |  |  |  |  |
| 3. Worrying too much about different things |  |  |  |  |
| 4. Trouble relaxing |  |  |  |  |
| 5. Being so restless that it is hard to sit still |  |  |  |  |
| 6. Becoming easily annoyed or irritable |  |  |  |  |
| 7. Feeling afraid, as if something awful might happen |  |  |  |  |
